# 3,4-Dimethyl-1*H*-1,2,4-triazepino[2,3-*a*]benzimidazol-2(3*H*)-one

**DOI:** 10.1107/S1600536810013498

**Published:** 2010-04-17

**Authors:** Asmae Saber, Abdusalam Al Subari, Hafid Zouihri, El Mokhtar Essassi, Seik Weng Ng

**Affiliations:** a Laboratoire de Chimie Organique Hétérocyclique, Pôle de Compétences Pharmacochimie, Université Mohammed V-Agdal, BP 1014 Avenue Ibn Batout, Rabat, Morocco; b CNRST Division UATRS, Angle Allal Fassi/FAR, BP 8027 Hay Riad, 10000 Rabat, Morocco; c Department of Chemistry, University of Malaya, 50603 Kuala Lumpur, Malaysia

## Abstract

In the mol­ecule of the title compound, C_12_H_12_N_4_O, a C atom and an N atom of the benzimidazole fused-ring portion are part of a seven-membered ring; this ring adopts a boat-shaped conformation (with the fused-ring atoms representing the stern and the *sp* ^3^-hybridized C atom the prow). The amino group is a hydrogen-bond donor to the imidazole group of an inversion-related mol­ecule, the pair of N—H⋯N hydrogen bonds giving rise to a hydrogen-bonded dimer.

## Related literature

For the synthesis, see: Romano *et al.* (1988 ▶).
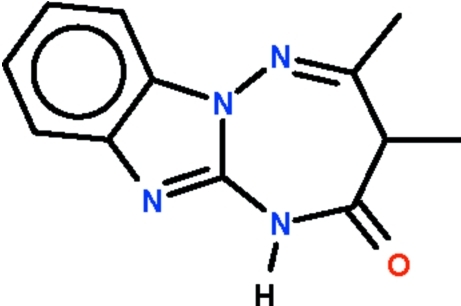


## Experimental

### 

#### Crystal data

C_12_H_12_N_4_O
*M* *_r_* = 228.26
Monoclinic, 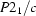

*a* = 7.2899 (3) Å
*b* = 14.4888 (5) Å
*c* = 10.9932 (4) Å
β = 104.314 (1)°
*V* = 1125.08 (7) Å^3^
*Z* = 4
Mo *K*α radiation
μ = 0.09 mm^−1^
*T* = 293 K
0.21 × 0.19 × 0.16 mm

#### Data collection

Bruker X8 APEXII diffractometer
14670 measured reflections
3095 independent reflections
1823 reflections with *I* > 2σ(*I*)
*R*_int_ = 0.034

#### Refinement

*R*[*F* ^2^ > 2σ(*F* ^2^)] = 0.049
*wR*(*F* ^2^) = 0.157
*S* = 1.01
3095 reflections
160 parameters
1 restraint
H atoms treated by a mixture of independent and constrained refinement
Δρ_max_ = 0.24 e Å^−3^
Δρ_min_ = −0.19 e Å^−3^

### 

Data collection: *APEX2* (Bruker, 2008 ▶); cell refinement: *SAINT* (Bruker, 2008 ▶); data reduction: *SAINT*; program(s) used to solve structure: *SHELXS97* (Sheldrick, 2008 ▶); program(s) used to refine structure: *SHELXL97* (Sheldrick, 2008 ▶); molecular graphics: *X-SEED* (Barbour, 2001 ▶); software used to prepare material for publication: *publCIF* (Westrip, 2010 ▶).

## Supplementary Material

Crystal structure: contains datablocks global, I. DOI: 10.1107/S1600536810013498/si2255sup1.cif

Structure factors: contains datablocks I. DOI: 10.1107/S1600536810013498/si2255Isup2.hkl

Additional supplementary materials:  crystallographic information; 3D view; checkCIF report

## Figures and Tables

**Table 1:** Hydrogen-bond geometry (Å, °)

*D*—H⋯*A*	*D*—H	H⋯*A*	*D*⋯*A*	*D*—H⋯*A*
N1—H1⋯N4^i^	0.86 (1)	2.01 (1)	2.867 (2)	174 (2)

Symmetry code: (i) 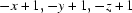.

**Fig. 1.**
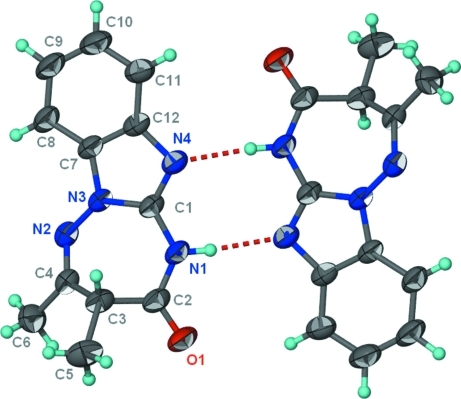
Thermal ellipsoid plot (Barbour, 2001) of the hydrogen-bonded dimeric structure of C12H12N4O at the 50% probability level; hydrogen atoms are drawn as arbitrary radius. Symmetry code for the inversion related molecule: 1 - x, 1 - y, 1 - z.

## supplementary crystallographic information

### Comment

1,2-Diaminobenzimidazoles react with β-dicarbonyl compounds to form 1,2,4-triazepino[2,3-*a*]benzimidazoles (Romano *et al.*, 1988), a class of compounds used in the treatment of neuronal disorders. The title compound (Scheme I, Fig. 1) was synthesized from 2-aminobenzimidazole and ethyl 2-methylacetoacetate. A carbon atom and a nitrogen atom of the benzimidazole fused-ring portion are part of a seven-membered ring; this ring adopts a boat-shaped conformation (with the fused-ring atoms representing the stern and the *sp*^3^-hybridized carbon atom the prow). Its methyl substitent occupies a quasi-equatorial position. The amino group is hydrogen-bond donor to the imidazole group (Table 1) of an inversion-related molecule, the pair of N—H···N hydrogen bonds (Fig. 1) giving rise to a hydrogen-bonded dimer.

### Experimental

2-Aminobenzimidazole (1 g, 6.75 mmol) and a slight excess of ethyl 2-methylacetoacetate (1.69 ml) were refluxed in xylene (10 ml) and acetic acid (0.5 ml) for 3 hours. The mixture was concentrated under reduced pressure and the resulting residue was recrystallized from ethanol. Brown crystals were isolated when the solvent was allowed to evaporate.

### Refinement

Carbon-bound H-atoms were placed in calculated positions (C—H 0.93–0.98 Å) and were included in the refinement in the riding model approximation, with *U*_iso_(H) set to 1.2*U*_eq_(C). The amino H-atom was located in a difference Fourier map, and was refined with a distance restraint of N–H 0.86 (1) Å.

### Crystal data

**Table:**

C_12_H_12_N_4_O	*F*(000) = 480
*M**_r_* = 228.26	*D*_x_ = 1.348 Mg m^−^^3^
Monoclinic, *P*2_1_/*c*	Mo *K*α radiation, λ = 0.71073 Å
Hall symbol: -P 2ybc	Cell parameters from 3188 reflections
*a* = 7.2899 (3) Å	θ = 2.4–24.8°
*b* = 14.4888 (5) Å	µ = 0.09 mm^−^^1^
*c* = 10.9932 (4) Å	*T* = 293 K
β = 104.314 (1)°	Prism, brown
*V* = 1125.08 (7) Å^3^	0.21 × 0.19 × 0.16 mm
*Z* = 4	

### Data collection

**Table:**

Bruker X8 APEXII diffractometer	1823 reflections with *I* > 2σ(*I*)
Radiation source: fine-focus sealed tube	*R*_int_ = 0.034
graphite	θ_max_ = 29.4°, θ_min_ = 2.4°
φ and ω scans	*h* = −9→10
14670 measured reflections	*k* = −20→19
3095 independent reflections	*l* = −15→13

### Refinement

**Table:**

Refinement on *F*^2^	Primary atom site location: structure-invariant direct methods
Least-squares matrix: full	Secondary atom site location: difference Fourier map
*R*[*F*^2^ > 2σ(*F*^2^)] = 0.049	Hydrogen site location: inferred from neighbouring sites
*wR*(*F*^2^) = 0.157	H atoms treated by a mixture of independent and constrained refinement
*S* = 1.01	*w* = 1/[σ^2^(*F*_o_^2^) + (0.0742*P*)^2^ + 0.1838*P*] where *P* = (*F*_o_^2^ + 2*F*_c_^2^)/3
3095 reflections	(Δ/σ)_max_ < 0.001
160 parameters	Δρ_max_ = 0.24 e Å^−^^3^
1 restraint	Δρ_min_ = −0.19 e Å^−^^3^

### Fractional atomic coordinates and isotropic or equivalent isotropic displacement parameters (Å^2^)

**Table:**

	*x*	*y*	*z*	*U*_iso_*/*U*_eq_	
O1	0.40956 (19)	0.51793 (12)	0.14398 (13)	0.0762 (5)	
N1	0.5626 (2)	0.53766 (12)	0.34574 (15)	0.0574 (4)	
H1	0.486 (3)	0.5002 (13)	0.3696 (19)	0.075 (7)*	
N2	0.95622 (19)	0.59291 (9)	0.32932 (14)	0.0508 (4)	
N3	0.86966 (19)	0.60627 (9)	0.42759 (13)	0.0475 (4)	
N4	0.6799 (2)	0.58224 (10)	0.55706 (13)	0.0552 (4)	
C1	0.6977 (2)	0.57641 (11)	0.44172 (16)	0.0488 (4)	
C2	0.5294 (2)	0.55903 (14)	0.22125 (17)	0.0569 (5)	
C3	0.6491 (2)	0.63705 (13)	0.18892 (16)	0.0551 (5)	
H3	0.6428	0.6886	0.2454	0.066*	
C4	0.8529 (2)	0.60475 (11)	0.21815 (17)	0.0498 (4)	
C5	0.5699 (3)	0.67076 (16)	0.05638 (19)	0.0797 (7)	
H5A	0.6472	0.7202	0.0387	0.120*	
H5B	0.4429	0.6926	0.0475	0.120*	
H5C	0.5692	0.6210	−0.0014	0.120*	
C6	0.9446 (3)	0.58710 (16)	0.11287 (19)	0.0694 (6)	
H6A	1.0719	0.5659	0.1463	0.104*	
H6B	0.9466	0.6432	0.0667	0.104*	
H6C	0.8742	0.5409	0.0580	0.104*	
C7	0.9748 (2)	0.62947 (10)	0.54769 (16)	0.0486 (4)	
C8	1.1602 (3)	0.65870 (13)	0.59037 (19)	0.0636 (5)	
H8	1.2399	0.6664	0.5369	0.076*	
C9	1.2194 (3)	0.67573 (16)	0.7180 (2)	0.0790 (7)	
H9	1.3432	0.6950	0.7517	0.095*	
C10	1.0993 (4)	0.66492 (16)	0.7969 (2)	0.0780 (7)	
H10	1.1444	0.6782	0.8819	0.094*	
C11	0.9147 (3)	0.63502 (14)	0.75350 (18)	0.0653 (5)	
H11	0.8348	0.6279	0.8070	0.078*	
C12	0.8542 (3)	0.61603 (11)	0.62561 (16)	0.0516 (4)	

### Atomic displacement parameters (Å^2^)

**Table:**

	*U*^11^	*U*^22^	*U*^33^	*U*^12^	*U*^13^	*U*^23^
O1	0.0480 (8)	0.1131 (12)	0.0627 (9)	−0.0193 (7)	0.0049 (6)	−0.0313 (8)
N1	0.0401 (8)	0.0756 (10)	0.0538 (9)	−0.0152 (7)	0.0068 (7)	−0.0171 (7)
N2	0.0385 (7)	0.0536 (8)	0.0580 (9)	−0.0022 (6)	0.0074 (7)	−0.0016 (6)
N3	0.0368 (7)	0.0521 (8)	0.0487 (8)	−0.0038 (6)	0.0012 (6)	−0.0036 (6)
N4	0.0461 (8)	0.0639 (9)	0.0520 (9)	−0.0080 (7)	0.0055 (7)	−0.0121 (7)
C1	0.0378 (8)	0.0540 (9)	0.0505 (9)	−0.0027 (7)	0.0035 (7)	−0.0103 (7)
C2	0.0349 (9)	0.0763 (12)	0.0565 (10)	0.0011 (8)	0.0056 (8)	−0.0202 (9)
C3	0.0468 (10)	0.0591 (10)	0.0536 (10)	0.0051 (8)	0.0014 (8)	−0.0080 (8)
C4	0.0404 (9)	0.0497 (9)	0.0563 (10)	−0.0035 (7)	0.0066 (8)	−0.0038 (7)
C5	0.0758 (15)	0.0797 (14)	0.0688 (13)	0.0114 (11)	−0.0103 (11)	−0.0030 (11)
C6	0.0578 (12)	0.0891 (15)	0.0640 (12)	−0.0009 (10)	0.0202 (10)	−0.0015 (10)
C7	0.0439 (9)	0.0417 (8)	0.0517 (9)	−0.0036 (7)	−0.0045 (8)	0.0010 (7)
C8	0.0482 (10)	0.0662 (11)	0.0657 (11)	−0.0128 (8)	−0.0065 (9)	0.0088 (9)
C9	0.0628 (13)	0.0825 (14)	0.0724 (14)	−0.0247 (11)	−0.0200 (11)	0.0067 (11)
C10	0.0816 (16)	0.0786 (14)	0.0575 (12)	−0.0199 (11)	−0.0137 (11)	−0.0014 (10)
C11	0.0708 (13)	0.0655 (11)	0.0521 (10)	−0.0082 (10)	0.0009 (9)	−0.0061 (8)
C12	0.0501 (10)	0.0467 (9)	0.0507 (9)	−0.0036 (7)	−0.0012 (8)	−0.0047 (7)

### Geometric parameters (Å, °)

**Table:**

O1—C2	1.213 (2)	C5—H5B	0.9600
N1—C2	1.365 (2)	C5—H5C	0.9600
N1—C1	1.373 (2)	C6—H6A	0.9600
N1—H1	0.864 (9)	C6—H6B	0.9600
N2—C4	1.279 (2)	C6—H6C	0.9600
N2—N3	1.393 (2)	C7—C8	1.383 (2)
N3—C1	1.371 (2)	C7—C12	1.385 (3)
N3—C7	1.394 (2)	C8—C9	1.384 (3)
N4—C1	1.309 (2)	C8—H8	0.9300
N4—C12	1.396 (2)	C9—C10	1.386 (3)
C2—C3	1.523 (3)	C9—H9	0.9300
C3—C5	1.510 (3)	C10—C11	1.382 (3)
C3—C4	1.515 (2)	C10—H10	0.9300
C3—H3	0.9800	C11—C12	1.393 (2)
C4—C6	1.495 (3)	C11—H11	0.9300
C5—H5A	0.9600		
			
C2—N1—C1	126.41 (17)	H5A—C5—H5C	109.5
C2—N1—H1	118.6 (14)	H5B—C5—H5C	109.5
C1—N1—H1	114.7 (14)	C4—C6—H6A	109.5
C4—N2—N3	116.74 (14)	C4—C6—H6B	109.5
C1—N3—N2	130.27 (13)	H6A—C6—H6B	109.5
C1—N3—C7	105.84 (14)	C4—C6—H6C	109.5
N2—N3—C7	121.30 (14)	H6A—C6—H6C	109.5
C1—N4—C12	104.53 (15)	H6B—C6—H6C	109.5
N4—C1—N3	113.68 (14)	C8—C7—C12	123.37 (17)
N4—C1—N1	123.18 (16)	C8—C7—N3	131.12 (19)
N3—C1—N1	122.98 (16)	C12—C7—N3	105.50 (14)
O1—C2—N1	120.60 (19)	C7—C8—C9	115.6 (2)
O1—C2—C3	123.76 (18)	C7—C8—H8	122.2
N1—C2—C3	115.63 (14)	C9—C8—H8	122.2
C5—C3—C4	115.20 (18)	C8—C9—C10	121.89 (19)
C5—C3—C2	111.16 (15)	C8—C9—H9	119.1
C4—C3—C2	108.05 (14)	C10—C9—H9	119.1
C5—C3—H3	107.4	C11—C10—C9	122.06 (19)
C4—C3—H3	107.4	C11—C10—H10	119.0
C2—C3—H3	107.4	C9—C10—H10	119.0
N2—C4—C6	116.53 (16)	C10—C11—C12	116.7 (2)
N2—C4—C3	124.01 (16)	C10—C11—H11	121.7
C6—C4—C3	119.45 (16)	C12—C11—H11	121.7
C3—C5—H5A	109.5	C7—C12—C11	120.37 (17)
C3—C5—H5B	109.5	C7—C12—N4	110.36 (15)
H5A—C5—H5B	109.5	C11—C12—N4	129.27 (19)
C3—C5—H5C	109.5		
			
C4—N2—N3—C1	45.3 (2)	C5—C3—C4—C6	−14.2 (2)
C4—N2—N3—C7	−155.63 (15)	C2—C3—C4—C6	110.73 (19)
C12—N4—C1—N3	−1.68 (19)	C1—N3—C7—C8	176.13 (18)
C12—N4—C1—N1	173.69 (17)	N2—N3—C7—C8	12.6 (3)
N2—N3—C1—N4	164.44 (16)	C1—N3—C7—C12	−2.84 (17)
C7—N3—C1—N4	2.94 (19)	N2—N3—C7—C12	−166.39 (14)
N2—N3—C1—N1	−10.9 (3)	C12—C7—C8—C9	−1.1 (3)
C7—N3—C1—N1	−172.44 (16)	N3—C7—C8—C9	−179.90 (18)
C2—N1—C1—N4	152.30 (18)	C7—C8—C9—C10	−0.6 (3)
C2—N1—C1—N3	−32.8 (3)	C8—C9—C10—C11	1.2 (4)
C1—N1—C2—O1	177.59 (18)	C9—C10—C11—C12	0.0 (3)
C1—N1—C2—C3	−3.3 (3)	C8—C7—C12—C11	2.3 (3)
O1—C2—C3—C5	13.2 (3)	N3—C7—C12—C11	−178.66 (16)
N1—C2—C3—C5	−165.91 (17)	C8—C7—C12—N4	−177.09 (16)
O1—C2—C3—C4	−114.2 (2)	N3—C7—C12—N4	1.99 (18)
N1—C2—C3—C4	66.8 (2)	C10—C11—C12—C7	−1.6 (3)
N3—N2—C4—C6	−177.91 (15)	C10—C11—C12—N4	177.58 (18)
N3—N2—C4—C3	3.5 (2)	C1—N4—C12—C7	−0.27 (19)
C5—C3—C4—N2	164.30 (17)	C1—N4—C12—C11	−179.55 (19)
C2—C3—C4—N2	−70.7 (2)		

### Hydrogen-bond geometry (Å, °)

**Table:**

*D*—H···*A*	*D*—H	H···*A*	*D*···*A*	*D*—H···*A*
N1—H1···N4^i^	0.86 (1)	2.01 (1)	2.867 (2)	174 (2)

Symmetry codes: (i) −*x*+1, −*y*+1, −*z*+1.
